# Subgroups of non-suicidal self-injury in a large diverse sample of online help-seekers

**DOI:** 10.3389/fpsyt.2025.1513685

**Published:** 2025-02-27

**Authors:** Kaylee P. Kruzan, Jason J. Washburn, David Aaby, Theresa Nguyen, David C. Mohr

**Affiliations:** ^1^ Department of Preventive Medicine, Northwestern University Feinberg School of Medicine, Chicago, IL, United States; ^2^ Department of Psychiatry and Behavioral Sciences, Northwestern University Feinberg School of Medicine, Chicago, IL, United States; ^3^ Mental Health America, Alexandria, VA, United States

**Keywords:** nonsuicidal self-injury, subgroups, latent class analysis, heterogeneity, subtypes, online help-seeking

## Abstract

**Introduction:**

Many young people access information and resources for nonsuicidal self-injury (NSSI) online; yet our understanding of who accesses such information is limited. NSSI is a behavior with varied presentations. Understanding heterogeneity can help guide person-centered intervention. The present study aimed to (1) empirically identify classes of individuals with NSSI and (2) compare the classes according to demographic and clinical characteristics.

**Methods:**

Data were collected from a survey posted to a national advocacy group website. Latent class analysis was used to derive classes based on characteristics associated with NSSI severity. Relationships between the latent classes and variables along five dimensions (behavior change, consequences or life interference, expectancies, functions, and NSSI across lifetime) were explored via logistic regression models.

**Results:**

11,262 individuals reporting past month NSSI were included in analyses. The 4-class model provided the most clinically interpretable groups. Class 1 was the smallest (16.8%), scored highest on all items and reported the youngest age of onset. Class 3 was the largest (31.8%), scored lowest on all items and reported the latest age of onset. Classes 2 (29.3%) and 4 (22.2%) had moderate scores on most items and differed in levels of suicidal ideation.

**Conclusion:**

Classes presented with more severe symptoms than what is typical in samples in extant literature underscoring the importance of tailoring interventions for dissemination in online contexts.

## 1 Introduction

Nonsuicidal self-injury (NSSI) is a complex behavior that affects around one in five young people (1, 2). While NSSI often signals underlying distress, efforts to identify, treat and intervene in the behavior are complicated by its varied presentations (3, 4). Extant research has shown significant heterogeneity among individuals that engage in NSSI (5). NSSI is often comorbid with other mental health conditions like borderline personality disorder and eating disorders (6, 7), but can also occur without other diagnoses (8). Additionally, individuals injure themselves in a variety of ways and can engage in behavior for many intrapersonal and interpersonal reasons (9, 10).

Some of the variability among individuals that engage in NSSI has been associated with more severe presentations of the behavior (11, 12). Methods that can result in greater tissue damage, the use of multiple methods, and some functions such as anti-dissociation or suicide prevention are common among individuals with more severe NSSI across many studies (13, 14). Further, although NSSI is enacted without suicidal intent many individuals that engage in NSSI also experience suicidal ideation (15, 16), which is another indicator of severity.

NSSI heterogeneity has been explored descriptively through consulting with individuals with lived experience and quantitatively with methods capable of deriving homogeneous subgroups like cluster analysis and latent class analysis. Qualitative studies have aimed to understand individual perceptions of the relationship between NSSI and suicide behavior (17), online activity (18, 19), NSSI motives (20), individual and social factors contributing to NSSI maintenance (21) and recovery (22). For example, Taliaferro et al. conducted an interview study exploring NSSI functions among 15 adolescents recently hospitalized for suicidal behavior. They found that while most adolescents engaged in NSSI to regulate negative emotions, how often they engaged in the behavior and what methods they used varied over time as they habituated to its effect (17). Miller et al., similarly explored reasons for NSSI in interview study with 9 adolescents engaged in treatment and found strong support for the emotion regulation function of the behavior, as well as NSSI as a means to prevent suicidal thoughts and behavior (20). While these studies have been valuable in understanding the behavior, and have direct implications for treatment, they have focused on small samples and have been less helpful to understand how certain characteristics of NSSI group together.

Data-driven approaches highlight heterogeneity in NSSI samples and relationships between NSSI characteristics. These studies commonly differentiate groups based on NSSI functions (23, 24), motives (25), methods (26, 27), and various psycho-social related factors. For example, several studies have found subgroups that report primarily interpersonal or intrapersonal functions for their NSSI behavior (28). Studies have also shown great variability in method and number of methods. For example, one study identified five subgroups that differed in method and method diversification, as well as frequency, functions, age of onset and gender (29). By contrast, Kim et al. (2023) found two groups that primarily differed in method – one that primarily used cutting and scratching and another that engaged in indirect self-harm.

Research exploring heterogeneity among individuals with NSSI is critical and can support the development of person-centered treatment and intervention. However, extant research has largely been conducted in small samples of relatively homogeneous groups (e.g., adolescent inpatients, undergraduate students). In addition, studies have typically focused on a limited number of NSSI characteristics e.g., motives, functions, frequency, method. To understand NSSI heterogeneity further research is needed in larger and more diverse populations that differ in socio-demographics as well as treatment history.

The aims of the current study were to (1) empirically identify classes of individuals with NSSI based on characteristics of NSSI and (2) compare the groups according to demographic and clinical characteristics.

## 2 Materials and methods

### 2.1 Participants and procedure

This study is part of a partnership with a leading national mental health advocacy group, Mental Health America (MHA, mhanational.org). MHA hosts screening tools for common mental health conditions on their website (e.g., PHQ-9, GAD-7). Any website visitor can complete the online screening tools. Data for this study come from an NSSI survey that was hosted on the website between December 2022 and May 2023 (See **Appendix A** in **Supplementary Materials** for full survey). The survey was developed as part of a broader project with the aim of developing a brief screening instrument. The survey included items relevant to the proposed DSM-5 criteria for NSSI disorder, as well as items to tap into constructs typically associated with more severe NSSI presentations and increased suicide risk such as age of onset (11) and function (30). Any visitor on MHA’s website could voluntarily take the survey, but only those reporting NSSI on one or more days in the past month were included in the present set of analyses. A total of 11,262 individuals were included in the analysis. All items on the NSSI survey were completed by all participants. A portion of participants completed an optional demographics survey following the NSSI survey.

The University Institutional Review Board deemed this research not human subjects because MHA was the site of recruitment and data collection, and data were fully de-identified (e.g., removal of IP addresses and other potentially identifying data) prior to secure transfer to the researchers for analysis. This study was not pre-registered.

### 2.2 Variables

Demographic information collected from the survey included age, gender, sexual orientation, race/ethnicity, and household income. We included 10 items from a larger survey assessing characteristics of NSSI in the LCA model to define classes. Two variables assessed NSSI over the lifetime (age of onset and extent of scarring), two variables assessing monthly and daily frequency of NSSI, and six variables that assessed frequency in the past month (urges, thoughts, severity of wounds, injuring while using substances, injuring more severely than intended, and suicidal ideation). Most items were collected on a 5-point Likert scale ranging from 1=never to 5=several times a day, except age of onset (less than 10; 10-12; 13-15; 16-17; 18 years or older) and extent of lifetime scarring (ranged from 1=never to 5=always). Monthly and daily frequency were collected as counts and transformed into categories based on quartiles for the LCA (Monthly: less than 3 days; 3 - 4 days, 5 - 11 days; 12 or more days; Daily: less than 2 times; 2 times, 3-4 times, 5 or more times)

Thirty-three dependent variables, distinct from the variables used in the LCA, assessed four dimensions: behavior change, consequences or life interference, expectancies and functions, and NSSI across lifetime. These included: habituation, desire for behavior change, consequences in various life domains (social, work/school, self-care and activities), duration of NSSI (Last month only, <3 months, < 6 months, 6-12 months, 1-2 years, more than 2 years), lifetime frequency, expectancies (to stop negative feelings, to induce good feelings, for social reasons), and functions (anti-dissociation and suicide prevention).

### 2.3 Statistical analysis

All analyses were conducted using Stata statistical software (31). We used latent class analysis (LCA) to empirically identify classes of participants who exhibited similar patterns of NSSI characteristics (32). The number of classes was selected using the Akaike information criterion (AIC); Schwarz Bayesian information criterion (BIC); Rissanen sample-size adjusted BIC; entropy, with higher values indicating better classification of individuals; and ease of interpretation (that is, the classes distinguished differences from a substantive perspective).

Maximum conditional probabilities for the indicator variables were used to characterize each class. Variables with probabilities greater than 0.50 were considered highly endorsed. Monthly and daily frequency were input as categorical variables based on quartiles. We used ordinal logistic regression (for Likert-scale outcomes) and ordinary logistic regression (for binary outcomes) to examine whether latent class membership was associated with different NSSI characteristics. Each characteristic was modeled separately, using the latent classes as predictors in the model. Differences between classes were evaluated using the pseudo class method, with 20 imputations (33). We chose the pseudo-class method because: (1) it provides conservative estimates of standard errors; and (2) it may perform optimally for models with moderate entropy (0.6) and competitively for models with large entropy (0.8). We present odds ratios (ORs) and 95% confidence intervals from the regression models. We also explored differences in classes by demographics (age, gender), NSSI expectancies and desire for behavior change. As a sensitivity analysis, we repeated logistic regression models for hypothesized relationships, adjusting for age and age of onset. Results were substantially similar. The age and age of onset adjusted model results are included **Supplementary Materials** (**Appendices C, D**).

There were no missing data for variables included in the NSSI survey as all questions were required to submit the form. The only missing data that came from the optional survey following the NSSI survey on MHA’s website, including sociodemographic and treatment variables: age (<1% missing), gender (1.8% missing) and lifetime treatment (5.5% missing), were low.

We expected to find at least two groups, though likely more given the sample size, that differed in the extent to which they endorsed items. Those with high endorsement of items would be classified as having higher severity, relative to those with low endorsement. Along with this, we expected to observe several relationships between classes and other characteristics commonly associated with more severe presentations of NSSI. Specifically, we expected that classes with higher NSSI severity would report engaging in NSSI for a longer duration (H1), greater lifetime frequency (H2), greater habituation (H3), more perceived life interference (H4), more often injuring in the past month with an anti-dissociation (H5a) and suicide prevention function (H5b). In addition, we expected that classes with higher NSSI severity would be more likely to report injuring on the face (H6a) and private parts of the body (H6b), greater trauma history (H7), and greater rates of lifetime (H8a) and current treatment (H8b), relative to those with lower severity.

## 3 Results

### 3.1 Sample characteristics

**Table 1** shows demographics and NSSI characteristics of the sample.

**Table 1:** Sample characteristics.

Characteristic	N = 11,262
Age (years) ^1^	17.1 (7.7)
Gender	
Female	7,402 (65.7%)
Male	2,037 (18.1%)
Non-Binary	1,621 (14.4%)
Missing	202 (1.8%)
Transgender^^^^	1,820 (16.2%)
Sexual Orientation	
Asexual	514 (8.7%)
Bisexual	2,090 (35.5%)
Lesbian or Gay	1,092 (18.6%)
Pansexual	1,292 (22.0%)
Queer	496 (8.4%)
Other	399 (6.8%)
Race and Ethnicity^^^	
American Indian or Alaska Native	350 (3.3%)
Asian	570 (5.4%)
Black or African American (non-Hispanic)	750 (7.1%)
Hispanic or Latino	1,519 (14.3%)
Middle Eastern or North African	81 (0.8%)
Native Hawaiian or other Pacific Islander	44 (0.4%)
White (non-Hispanic)	5,925 (55.7%)
Other	380 (3.6%)
More than one of the above	1,011 (9.5%)
Household Income	
Less than $20,000	1,953 (17.3%)
$20,000 - $39,999	1,431 (12.7%)
$40,000 - $59,999	1,141 (10.1%)
$60,000 - $79,999	878 (7.8%)
$80,000 - $99,999	628 (5.6%)
$100,000 - $149,999	699 (6.2%)
$150,000+	628 (5.6%)
Missing	3,904 (34.7%)
Lifetime Treatment	5,383 (50.6%)
Current Treatment	3,123 (58.9%)
Monthly frequency^1^	8.2 (8.4)
Day frequency^1^	4.2 (8.1)

Mean (SD); n (%).

*Unless otherwise noted questions were answered the full sample (n = 16,733).

^Race and ethnicity are reported together in line with the way data were collected on the [redacted] website. Participants were asked their race and ethnicity via a single question with nine mutually exclusive response options.

^^Identifying as transgender was assessed in a separate item and not counted towards the overall *n* in Gender

### 3.2 Determination of latent classes

**Table 2** displays the results of latent class analyses for one to eight classes. We determined that the 4-class model was optimal (AIC 62528; adjusted BIC 63171; entropy 0.74). Although the AIC and adjusted BIC continued to decrease as we increased the number of classes, the entropy remained stable. The increased number of classes did not differ in any meaningful or interpretable way. The 4-class model provided the most clinically interpretable groups.

**Table 2:** Model fit statistics for latent class analysis specifying 1-8 class solutions.

Classes	AIC	BIC	Adj BIC	Entropy
1	84299.75	84578.26	84457.5	1
2	69010.2	69574.55	69329.85	0.7611738
3	64850.09	65700.27	65331.64	0.7601656
**4**	**62527.71**	**63663.74**	**63171.17**	**0.7376905**
5	60870.09	62291.95	61675.44	0.7390079
6	59998.69	61706.39	60965.95	0.7385087
7	59594.23	61587.77	60723.39	0.7188645
8	59140.45	61419.82	60431.51	0.7087434

The bolded represents the selected model.

*AIC*, Akaike’s Information Criterion; *BIC*, Bayesian Information Criterion; *Adj BIC*, Rissanen sample-size adjusted BIC.

### 3.3 Characteristics of classes

**Table 3** shows descriptives of the variables that went into the classes. Class 1 was the smallest in the sample (16.8%) and labeled “High NSSI severity” because members of this class scored highest on all items and reported the youngest age of onset. By contrast, Class 3 was the largest (31.8%) and labeled “Low NSSI severity.” Members of this class scored lowest on all items and reported the latest age of onset. Class 2 (29.3%) and Class 4 (22.2%) had moderate scores on most items; however, Class 2 was characterized by frequent suicidal ideation and was labeled “Moderate NSSI severity - High suicidal ideation.” Thus, class 4 was labeled “Moderate NSSI severity.”

**Table 3:** Characteristics by latent classes.

Characteristic	Class 1: High NSSI Severity N = 1,870	Class 2: Moderate NSSI severity with High Suicidal Ideation N = 3,385	Class 3: Low NSSI Severity N = 3,630	Class 4: Moderate NSSI Severity N = 2,377
Age of Onset				
Less than 10 years old	718 (38%)	542 (16%)	348 (9.6%)	491 (21%)
10-12 years old	859 (46%)	1,560 (46%)	1,239 (34%)	1,234 (52%)
13-15 years old	243 (13%)	975 (29%)	1,370 (38%)	543 (23%)
16-17 years old	20 (1.1%)	176 (5.2%)	343 (9.4%)	52 (2.2%)
18 years or older	30 (1.6%)	132 (3.9%)	330 (9.1%)	57 (2.4%)
Number of Days	17 (10)	8 (8)	3 (4)	10 (8)
Times per Day	9 (15)	3 (6)	2 (3)	4 (7)
Permanent Scars^1^	3.70 (1.05)	2.84 (1.20)	2.36 (1.16)	3.04 (1.02)
Urge^2^	4.86 (0.45)	4.68 (0.53)	3.03 (0.88)	3.90 (0.71)
Thoughts^2^	4.80 (0.53)	4.83 (0.43)	3.11 (1.02)	3.70 (0.84)
Self Care^2^	3.20 (1.31)	1.23 (0.52)	1.05 (0.25)	1.82 (0.87)
Substance^2^	1.89 (1.38)	1.25 (0.65)	1.17 (0.52)	1.42 (0.84)
Severe^2^	3.57 (1.13)	1.58 (0.66)	1.29 (0.49)	2.34 (0.80)
Suicide^2^	4.68 (0.74)	4.32 (1.04)	2.66 (1.25)	3.35 (1.17)

n (%); Mean (SD).

All items were asked within the past month except Age of Onset.

^1^Response categories were: 1 (Never), 2 (Sometimes), 3 (About half the time), 4 (Most times), 5 (Always).

^2^Response categories were: 1 (Never), 2 (Once or twice during the past 30 days), 3 (Around once a week), 4 (Several times a week), 5 (Several times a day).

Members of the High NSSI severity class were the youngest on average (*M*=13, *IQR*=12, 15), followed by the Moderate NSSI severity class (*M*=14, *IQR*=13, 16), the Moderate NSSI Severity - High suicidal ideation class (*M*=15, *IQR*=13, 18), and the Low NSSI severity class (*M*=16, *IQR*=14, 21). Classes were similar by gender. The majority of individuals were female (67%), followed by male (18%), and non-binary (15%).

Most members in the High NSSI severity class reported beginning NSSI before 12 years-old (83.8%), with 38.1% engaging in the behavior before age 10, and 45.7% between the ages of 10 and 12 years-old. Members of this class reported the most frequent past monthly and daily NSSI and the highest rates of injuring themselves more severely than intended (24.7% reporting doing so several times a day – compared to <1% in all other classes) and being unable to care for their wounds alone (23.4% several times a day compared to <=1% in all other classes). They also more strongly endorsed lifetime scarring (25.3% reporting that their injuries always resulted in scarring, as compared to <10% in all other classes). The vast majority of members in this class (93.2%) reported having thoughts of suicide at least several times a week (compared to 84.3% in the Moderate NSSI severity - High suicidal ideation class, 51.6% in the Moderate NSSI severity class and 30.2% in the Low NSSI severity class).

Members in the Moderate NSSI severity and the Moderate NSSI severity - High suicidal ideation classes reported similar patterns in their age of onset with most members reporting initiating NSSI behaviors between 10-12 years old, followed by 13-15 years old, and lastly before 10 years old. Members of the moderate classes reported similar rates of lifetime scarring (numbers with roughly 9% in both classes reporting leaving scars always) and injuring while under the influence of substances (between 75 - 82% reporting never).

While these classes had some similarities they differed in their rates of suicidal ideation. Members of the Moderate NSSI severity - High suicidal ideation class reported much higher rates of suicidal ideation with the majority (58%) reporting having suicidal thoughts several times a day, 25% reporting suicidal thoughts several times a week and just 7% reporting suicidal ideation once a week or less. By contrast, 19% of those in the Moderate NSSI severity class reported daily suicidal thoughts, followed by 32% reporting weekly suicidal ideation, and 48% reporting suicidal ideation once a week or less. Several additional differences between these classes were apparent. The Moderate NSSI severity-High Suicidal Ideation group reported more frequent NSSI thoughts and urges – with rates more similar to the High NSSI severity class. Relative to the Moderate NSSI severity group, the Moderate NSSI severity - High suicidal ideation class also reported less frequent past month and daily NSSI, fewer instances of needing help to care for wounds (78.5% reporting never as compared to 43.9%) and fewer reports of injuring more severely than intended (49.9% reporting never as compared to 13.6%).

Finally, the majority of members in the Low NSSI severity class reported age of onset between 10-15 years old, with less than 10% of members in this class reported beginning NSSI prior to age 10. Members of this class reported the least frequent past month and daily NSSI and the lowest rates on all other items. The majority reported never injuring themselves more severely than intended (70.7%), never being unable to care for their wounds alone (94%), and infrequently injuring in ways that caused scarring (63% reporting never or sometimes, compared to 16% in the High NSSI severity class).

### 3.4 Comparisons of latent classes

Pairwise comparisons of latent classes with odds ratios are depicted in **Appendix B** of **Supplementary Materials**.

#### 3.4.1 Hypothesis testing

In support of our first two hypotheses, the classes with higher NSSI severity were at greater odds of engaging in NSSI for a longer duration (H1) and reporting higher lifetime NSSI frequency (H2). For example, compared to the Low NSSI severity, those in the High, Moderate - High suicidal ideation and Moderate classes had 1.67, 1.33, and 1.25 times the odds of an increased level of duration, respectively (class 1 vs 3: 95% CI 1.49, 1.87; class 2 vs 3: 95% CI 1.20, 1.47; class 4 vs 3: 95% CI 1.12, 1.40). Over half of those in the High NSSI severity class (53%) reported engaging in NSSI for more than 2 years, as compared to 47% in the Moderate NSSI severity - High suicidal ideation class, 44% in the Moderate NSSI severity class and 40% in the Low NSSI severity class. Similarly, half of the members of the High NSSI severity class reported injuring themselves more than 50 times in their lifetime, as compared to 37% in the Moderate NSSI severity - High suicidal ideation class, 33% in the Moderate NSSI severity class, and 18% in the Low NSSI severity class. The classes were more similarly distributed across the other response categories.

In support of H3, classes with greater NSSI severity had greater odds of endorsing habituation. The High NSSI severity class was at nearly 10 times the odds of strongly endorsing habituation to NSSI, relative to the Low NSSI severity class (OR 9.75; 95% CI 8.60, 11.06). Similarly, the Moderate NSSI severity - High suicidal ideation class (OR 2.96; 95% CI 2.67, 3.27) and the Moderate NSSI severity class (OR 3.22; 95% CI 2.88, 3.6) have 3 times the odds of reporting habituation compared to the Low NSSI severity class (OR 2.96; 95% CI 2.67, 3.27).

Consistent with H4, relative to the Low NSSI severity class the High NSSI severity class had 3-8 times the odds of reporting that their NSSI behaviors had interfered with functioning in other areas of life, relative to other classes. For example, compared to the Low NSSI severity class, the High NSSI severity class had 6.83 times the odds of increasing agreement that their NSSI behaviors affected their school/work tasks (95% CI 6.07, 7.68). The moderate classes had between 1.5 and 2 times the odds of reporting that NSSI interfered with other areas of life functioning relative to the low class. However, members of the Moderate NSSI severity class had greater odds of agreeing that NSSI interfered with life functioning, relative to the Moderate NSSI severity -High suicidal ideation class.

Consistent with our expectations on NSSI functions, the High NSSI severity class had over 4 times the odds of strongly endorsing engaging in NSSI for anti-dissociation (OR 4.27; 95% CI 3.80, 4.80) (H5a) and to prevent suicide (OR 4.33; 95% CI 3.82, 4.91), compared with the Low NSSI severity class (H5b). The moderate classes were similarly at increased odds of reporting these functions, when compared to the low class. There were not significant differences in endorsement of these functions between the moderate classes, however.

Consistent with H6, the High NSSI severity class was 1.69 times more likely to report injuring on their face (95% CI 1.48, 1.94) (H6a), 3.70 times more likely to report injuring private parts (95% CI 2.96, 4.63) (H6b), relative to the Low NSSI severity class. The moderate classes were at increased odds as well but only significantly differed from one another on their odds of injuring on the face.

When compared to all other classes the High NSSI severity class was at increased odds of experiencing all forms of trauma (H7). Like the patterns noted above, the moderate classes were at increased odds of reporting trauma except for intimate partner violence. There were no significant differences in trauma history between the moderate classes.

In contrast to our hypotheses regarding treatment, the High NSSI severity class had the lowest proportion of individuals reporting lifetime treatment (43%), followed by the Moderate NSSI severity class (49%), the Moderate NSSI severity - High suicidal ideation class (52%) and the Low NSSI severity class (54%) (H8a). Differences in the Moderate classes and the Moderate - High suicidal ideation class as compared to the Low NSSI severity class, were not statistically significant, however. Finally, there was no relationship observed between groups and current treatment (H8b).

#### 3.4.2 Other characteristics: expectancies and behavior change

In terms of expectancies, the High NSSI severity class had over 4 times the odds of endorsing engaging in NSSI to “feel good or better” (OR 4.63; 95% CI 4.11, 5.21) or to “help resolve problems with others,” (OR 4.56; 95% CI 4.05, 5.12) and 2.6 times more likely to endorse injuring to “stop or relieve bad feelings or thoughts,” (95% CI 2.30, 2.94) when compared to the Low NSSI severity class. The Moderate groups were at 1.4-2 times the odds of endorsing all expectancies, relative to the Low NSSI severity group. There were also slight differences in the moderate groups, when compared to each other such that the Moderate NSSI severity - High suicidal ideation class was at slightly increased odds of endorsing injuring “to feel good,” whereas the Moderate NSSI severity group was at increased odds of endorsing injuring for social purposes. There was no difference between the moderate groups in stopping bad feelings or thoughts.

Finally, interesting patterns emerged in the behavior change items. The High NSSI severity class had 0.35 times the odds of reporting wanting to decrease their behavior (95% CI 0.31, 0.39) and had 0.33 times the odds of reporting wanting to stop their NSSI behavior (95% CI 0.30, 0.37), relative to the low NSSI severity class. Yet, the High class had 1.31 times the odds of reporting that they “want to stop, but haven’t been able to.” (95% CI 1.17-1.48) A similar pattern was observed for the Moderate class, when compared to Low. When compared to the Moderate NSSI severity - High suicidal ideation class, however they were at increased odds of high endorsing all behavior change items.

## 4 Discussion

In this study, we sought to identify groups of individuals reporting past month engagement in NSSI. We did so in a large and diverse sample of individuals that sought out an online mental health screening through a national advocacy group’s website. Given this context, individuals may have been more aware of, or motivated to seek help and information about their mental health and NSSI behavior compared to other individuals that engage in NSSI. Notably, our sample reported relatively severe presentations of NSSI. Indeed, the sample looks more similar to clinical samples than college or community samples. For example, the High NSSI severity class reported past month rates of NSSI that are nearly three times higher than a recent study of adolescent inpatients (34).

Regarding our hypotheses, we found four classes that differed in ways consistent with the literature, as evidenced by support for most of our hypotheses. The class with the highest NSSI severity reported engaging in the behavior for the longest duration (H1), the highest lifetime NSSI frequency (H2), the greatest habituation to the behavior (H3), and the most perceived consequences or life interference because of the behavior (H4). Higher severity classes were also associated with greater endorsement of the anti-dissociative (H5a) and suicide prevention functions (H5b), were at increased odds of injuring on the face (H6a) and private parts of the body (H6b), and at increased odds of experiencing all forms of traumatic experiences (H7). While other studies have also found a high severity group in their samples (35, 36), in contrast to our expectations, the High NSSI severity class had the lowest proportion of individuals reporting lifetime treatment (H8a). Further, despite their clearly high need, there was no relationship between classes and current treatment (H8b).

The finding that individuals with the most severe presentations of NSSI were least likely to have a history of treatment is noteworthy. Less than one quarter of these individuals reported being in mental health treatment at the time they took the screening. This finding is in contrast to prior research that indicates more severe classes of NSSI are more likely to have had treatment (37). This discrepancy with the existing literature may be a function of the characteristics of the sample. While individuals with severe NSSI that are engaged in treatment may have little reason to visit an online mental health screening platform, those who are not in treatment may specifically seek out an anonymous screening to better understand their severe mental health difficulties. As such, this study may be identifying a unique subsample of individuals who engage in NSSI that is not found in studies that sample from school, college, or clinical populations. Indeed, past work similarly shows that individuals that sought mental health help online were younger, reported more frequent and recent NSSI behavior, and endorsed more suicidal thoughts when compared to individuals that had not sought help online (38). Literature has suggested that online spaces may be particularly compelling for individuals with stigmatized conditions due to their ability to maintain help-seeker’s anonymity and privacy (39, 40). Given many misunderstandings about NSSI behaviors by the public and professionals alike, online spaces may feel like a safer entry point to help.

It will be critical for future research to understand the needs of individuals with higher severity NSSI who are not in treatment, particularly barriers to treatment. There also may be a unique opportunity for online platforms to engage individuals who are seeking information about their NSSI to not only screen for NSSI, but to assess the severity of their NSSI to deliver early interventions for this high risk group.

The classes identified in this sample share similarities with other groups identified through other LCA studies. For example, our High NSSI severity class is similar to the Severe NSSI class identified in Case et al., 2020. Specifically, Case et al., identified four groups that differed in NSSI functions and other characteristics. Like our High NSSI severity class, Case’s Severe NSSI class reported high lifetime and last year NSSI frequency rates, high scar presence, and anti-suicide and anti-dissociation functions. Although not evaluated by Case et al. (2020), relative to all other classes our Severe NSSI class also had substantially more members reporting habituation and the highest levels of suicidal ideation. This finding aligns with Joiner’s IPTS, which suggests that repeated habituation to pain caused by NSSI may result in more intense injuries and greater tissue damage over time. Habituation and diminished aversion to injuring oneself is then thought to increase capacity for suicide and places individual at higher risk of engaging in suicidal behavior.

Importantly, the High NSSI severity class was the youngest on average but had engaged in the behavior for the longest period and had the earliest age of onset. From a developmental perspective, early adolescence is a period of heightened sensitivity to socio-affective pain and reward and less capacity to control impulses (41), which may put them at particularly risk of engaging in risky NSSI behaviors and practices (42). The younger age of the most severe NSSI class may also indicate a population of youth whose NSSI has not yet come to the attention of adults who may encourage or pressure youth into treatment. For these youth, access to anonymous online resources and supports may be especially important.

Members of the High NSSI severity class were the least likely to report wanting to stop or decrease their behavior, but the most likely to report that they want to stop but haven’t been able to. This aligns with literature on the functional and reinforcing qualities of NSSI (43, 44), and may make members of this class experience more ambivalence towards their behavior. This finding has direct implications for intervention, suggesting the need to prioritize and target motivation to change NSSI through strategies such as articulating the pros and cons of stopping NSSI. Given that these individuals were involved in help-seeking online, but the majority are not currently in treatment, it may also be that they are interested in changing their behavior or finding relief but have had limited success in doing so without support. Alternative regulatory strategies and psychoeducation on neuroplasticity to increase one’s belief in their ability to change, as has been done in single session interventions (45), may also be worthwhile approaches.

Similar to other studies (46, 47), our findings indicate that the majority of individuals who engage in NSSI have moderate levels of severity, but are split between on the basis of suicide severity. Differences in our moderate classes, however, extended beyond suicidal ideation and in ways that were unexpected. For example, the Moderate NSSI severity - High suicidal ideation class reported injuring less frequently and for a shorter duration, but had more frequent thoughts and urges, relative to the Moderate NSSI severity class. This class reported less life interference and endorsed wanting to end the behavior less strongly. The Moderate NSSI severity class reported less cognitive symptoms and more frequent behavior over a longer period. They were also more inclined to express wanting to change their behavior.

The differences in characteristics of NSSI found between the groups can inform treatment by personalizing intervention planning. Cognitive-behavioral and related third-wave approaches have the strongest evidence in the treatment of NSSI (48, 49), yet the degree to which cognitive or behavioral techniques are emphasized could vary. For example, for individuals in the moderate class with high suicidal ideation, a therapeutic approach that uses cognitive strategies to address NSSI thoughts and urges may be beneficial. In contrast, the moderate group with lower suicidal ideation and more frequent NSSI behaviors may benefit more from self-monitoring and behavioral strategies that change antecedents and consequences to reduce NSSI behavior.

Finally, when compared to prior studies, our Low NSSI Severity class has higher rates of lifetime NSSI on average than Mild/Experimental groups (37, 50), but shares similarly low suicidal ideation and tissue damage. This finding again suggests that this sample, on average, has a more severe presentation of NSSI than those typical in extant research that has examined clinical, college, or school populations. This group also reports engaging in NSSI for the shortest period, has the oldest age of onset, and the most desire to end the behavior. Members of this class may be highly motivated to change their behavior and may respond well to brief or even singular interventions such as online psychoeducation on NSSI and emotion regulation strategies.

### 4.1 Limitations

While this study has several strengths, findings must be considered with the following limitations in mind. First, individuals in our study were engaged with an online mental health resource and may represent a subgroup of the population with a greater awareness of their NSSI behavior and/or a greater motivation to change their behavior. Findings may not be generalizable to individuals less inclined to seek out mental health information online. Second, this study is cross-sectional, so we are not able to comment on how these characteristics are related to one another causally. Third, findings are based on self-report so there may have been recall bias in reflecting on frequencies of lifetime variables. Fourth, data were collected from an online survey and as such there may be sampling and response biases, including potential for fraudulent responses (51). While efforts were made to reduce the likelihood of fraudulent responses before data were transferred to the research team such as identifying and removing surveys that took less than one minute to complete or cases where multiple surveys were taken from the same IP address, it is still possible given the size of the sample that some fraudulent responses were missed. Finally, there are other characteristics relevant to NSSI severity that were not explored here. Future research should explore replication of these classes and extend the present findings through examining class relations with other characteristics commonly associated with clinical NSSI.

### 4.2 Conclusion

In this study we explored NSSI heterogeneity in a large and diverse sample of online help-seekers. We found four classes that varied on most NSSI characteristics related to severity and impact of the behavior. Our sample, on average, presented with more severe symptoms than what is typical in university or community samples underscoring the value of developing resources and interventions that can be easily disseminated online.

## Data Availability

The data that support the findings of this study are available from the corresponding author upon reasonable request.
