# Supplementary material for: Subgroups of non-suicidal self-injury in a large diverse sample of online help-seekers
Source: Front Psychiatry. 2025 Feb 27;16:1513685. doi: 10.3389/fpsyt.2025.1513685 (PMC11903480; doi:10.3389/fpsyt.2025.1513685)
Supplement: Supplementary file 1 [file Table1.docx]

Supplementary materials

Appendix A. NSSI survey

**About the self-injury survey**

In this survey, we want to learn more about times when ***you’ve hurt yourself on purpose without wanting to die***. When we say self-injury, we mean doing something that causes **immediate pain** or **physical injury**.

● **Self-injury** includes things like cutting, burning, or scratching your skin.

● Self-injury does **not** include things that don’t cause immediate pain, like eating too much or too little, using drugs or alcohol, or over-exercising.

● Self-injury is also **not** things that cause immediate pain but are socially acceptable like getting piercings and tattoos.

Our survey has between 16 - 26 questions. We wrote these questions to learn more about people’s experiences with self-injury.

**Why we are asking these questions.**

Answering these questions can help you to reflect on your experience of self-injury and will help us to do two things:

● Develop a test to help youth understand the amount of risk they have based on their current self-injury behaviors

● Create resources to help youth who self-injure and visit our site.

Some of these questions are sensitive. We are hoping to explore how people self injure and what the impact of self-injury is.

Some questions are similar, since we’re still in the process of choosing the best questions to explore this issue.

Questions may also be hard to answer because you may not remember specifics. Don’t think too much - just give your best guess!

Let’s get started.

Have you ever hurt yourself (engaged in self-injury) on purpose? Yes / No

**Self-injury survey**

Can you provide a few sentences about how you engage in self injury. You can write anything that feels right for you. (field text)

**Your history with self-injury.**

1. How old were you when you **first** engaged in self-injury?

● <10 years old

● 10-12 years old

● 13-15 years old

● 16-17 years old

● 18 years old or older

1. How old are you now?

● *______*

1. How **long** have you engaged in self-injury?

● Last month only

● <3 months

● < 6 months

● 6-12 months

● 1-2 years

● more than 2 years

1. About how many times in your life have you engaged in self-injury? **by “times” we are referring to an episode of self-injury. You may have multiple episodes in a day and multiple injuries in one episode.*

● 1 time

● 2-5 times

● 6-20 times

● 21-50 times

● more than 50 times

1. If you had to estimate, **how often** would you say that your injuries resulted in permanent scars?

● Never

● Sometimes

● About half the time

● Most times

● Always

**In the following questions, please think about your self-injury behaviors in the past month (30 days).**

1. In the past month, on how many **days** have you engaged in self-injury?

● 0-30 days

1. On the days that you engaged in self-injury, how many **times per day** did you hurt yourself?

● 0-100 times per day

1. In the past month, how often have you had an **urge (or a strong desire)** to injure yourself?

● Never

● Once or twice during the past 30 days

● Around once a week

● Several times a week

● Several times a day

1. In the past month, how often have you **thought about (even if it was a passing thought)** injuring yourself in the future?

● Never

● Once or twice during the past 30 days

● Around once a week

● Several times a week

● Several times a day

1. In the past month, how often have you injured yourself so badly you weren’t sure you could care for your wounds without help?

● Never

● Once or twice during the past 30 days

● Around once a week

● Several times a week

● Several times a day

1. In the past month, how often have you injured yourself when **using** drugs or alcohol?

● Never

● Once or twice during the past 30 days

● Around once a week

● Several times a week

● Several times a day

1. In the past month, how often have you injured yourself **more severely than you meant to**?

● Never

● Once or twice during the past 30 days

● Around once a week

● Several times a week

● Several times a day

1. In the past month, how often did you have negative feelings or thoughts (anger, sadness, self-criticism, etc.) *before injuring yourself?*

● Never

● Once or twice during the past 30 days

● Around once a week

● Several times a week

● Several times a day

1. In the past month, how often were you thinking or worrying about the idea of injuring yourself *before injuring yourself?*

● Never

● Once or twice during the past 30 days

● Around once a week

● Several times a week

● Several times a day

1. In the past month, how often have you experienced conflict or problems with other people *before injuring yourself?*

● Never

● Once or twice during the past 30 days

● Around once a week

● Several times a week

● Several times a day

1. In the past month, how often have you had thoughts of killing yourself or ending your life?

● Never

● Once or twice in the past 30 days

● Around once a week

● Several times a week

● Several times a day

**When thinking about your self-injury in the past month, please rate how much you agree or disagree with these statements.**

1. When I injure myself, I *expect it will* stop or relieve bad feelings or thoughts.

● Strongly disagree

● Disagree

● Unsure

● Agree

● Strongly agree

1. When I injure myself, *I expect it will* make me feel good, or better than I do.

● Strongly disagree

● Disagree

● Unsure

● Agree

● Strongly agree

1. When I injure myself, *I expect it will* help me resolve problems with others.

● Strongly disagree

● Disagree

● Unsure

● Agree

● Strongly agree

1. I injure myself to feel something when I’m numb or detached.

● Strongly disagree

● Disagree

● Unsure

● Agree

● Strongly agree

1. I injure myself to deal with, or prevent, thoughts or feeling of wanting to die or kill myself.

● Strongly disagree

● Disagree

● Unsure

● Agree

● Strongly agree

1. Over time, I have had to injure myself more deeply or in more places to get the same effect.

● Strongly disagree

● Disagree

● Unsure

● Agree

● Strongly agree

1. I want to decrease my self-injury.

● Strongly disagree

● Disagree

● Unsure

● Agree

● Strongly agree

1. I want to stop my self-injury.

● Strongly disagree

● Disagree

● Unsure

● Agree

● Strongly agree

1. I want to stop my self-injury, but haven’t been able to.

● Strongly disagree

● Disagree

● Unsure

● Agree

● Strongly agree

1. Self-injury has affected relationships that are important to me.

● Strongly disagree

● Disagree

● Unsure

● Agree

● Strongly agree

1. Self-injury has affected my ability to do school work or finish work tasks.

● Strongly disagree

● Disagree

● Unsure

● Agree

● Strongly agree

1. Self-injury has affected my ability to take care of myself.

● Strongly disagree

● Disagree

● Unsure

● Agree

● Strongly agree

1. Self-injury has affected my ability to do things that I like.

● Strongly disagree

● Disagree

● Unsure

● Agree

● Strongly agree

1. Please select all areas of the body where you’ve hurt yourself:

● Arms

● Chest

● Stomach or abdomen

● Face

● Legs

● Hand

● Feet

● Private parts

● Other ____

**Result page for initial deployment**

Thank you for answering these questions. The goal of this survey is to learn more about people’s experiences of self-injury to develop a test to help youth understand the amount of risk they have based on their current self-injury behaviors and create resources to help youth who self-injure and visit our site. It will also help us create resources to help visitors to this website.

Self-injury can feel like a lonely experience, but help is available. If you need immediate help, you can reach the Suicide & Crisis Lifeline by calling or texting 988 or using the chat box at [988lifeline.org](http://988lifeline.org/). You can also text "REDACTED" to 741-741 to reach the Crisis Text Line. [Warmlines](https://screening.mhanational.org/content/need-talk-someone-warmlines/) are an excellent place for non-crisis support.

Sometimes the first step in feeling better is simply learning more about your mental health. The articles below can help with some common feelings and challenges among people who self-harm:

[How can I tell someone I'm harming myself?](https://screening.mhanational.org/content/how-can-i-tell-someone-im-harming-myself/?layout=actions_neutral&ref=110%3E)

[How do I stop harming myself?](https://screening.mhanational.org/content/how-do-i-stop-harming-myself/?layout=actions_neutral&ref=110%3E)

[I don’t want to live, but I don’t want to die.](https://screening.mhanational.org/content/i-dont-want-live-i-dont-want-die/?layout=actions_neutral&ref=110%3E)

See all articles about [self-harm](https://screening.mhanational.org/self-harm/)

Self-harm often goes along with other mental health challenges. It may be helpful to take some of our other [mental health tests](https://screening.mhanational.org/screening-tools/). Self-harm is especially common among people dealing with [depression](https://screening.mhanational.org/screening-tools/depression/?ref), [anxiety](https://screening.mhanational.org/screening-tools/anxiety/?ref), [bipolar disorder](https://screening.mhanational.org/screening-tools/bipolar/?ref), and [addiction](https://screening.mhanational.org/screening-tools/addiction/?ref).

Appendix B. Pairwise comparisons and odds ratios for hypothesized relationships controlling for age of onset

|  | **High vs. Low** | **Moderate-SI vs. Low** | **Moderate vs. Low** | **High vs. Moderate-SI** | **Moderate vs. Moderate-SI** | **High vs. Moderate** |
| --- | --- | --- | --- | --- | --- | --- |
| **Hypothesized Relationships** |  |  |  |  |  |  |
| Duration (H1) | 1.16; (1.02, 1.31) | **1.2; (1.08, 1.33)** | 1.06; (0.95, 1.19) | 0.96; (0.85, 1.09) | 0.88; (0.78, 1) | 1.09; (0.96, 1.24) |
| Lifetime frequency (H2) | **2.98; (2.64, 3.35)** | **2.07; (1.88, 2.28)** | **1.78; (1.59, 1.99)** | **1.44; (1.28, 1.62)** | **0.86; (0.77, 0.96)** | **1.67; (1.47, 1.9)** |
| Habituation (H3) | **8.77; (7.69, 10.01)** | **2.8; (2.53, 3.1)** | **2.98; (2.66, 3.34)** | **3.13; (2.73, 3.6)** | 1.06; (0.94, 1.2) | **2.94; (2.57, 3.36)** |
| Consequences/Life interference (H4) |  |  |  |  |  |  |
| *Enjoyable activities* | **6.45; (5.67, 7.34)** | **2.17; (1.97, 2.39)** | **2.47; (2.22, 2.74)** | **2.98; (2.63, 3.37)** | 1.14; (1.02, 1.27) | **2.62; (2.31, 2.96)** |
| *Relationships* | **3.34; (2.96, 3.76)** | **1.54; (1.39, 1.7)** | **1.91; (1.71, 2.14)** | **2.17; (1.94, 2.42)** | **1.24; (1.12, 1.38)** | **1.75; (1.54, 1.98)** |
| *Ability to care for self* | **7.25; (6.4, 8.2)** | **2.15; (1.96, 2.36)** | **2.48; (2.23, 2.76)** | **3.37; (2.97, 3.83)** | **1.15; (1.04, 1.28)** | **2.92; (2.57, 3.32)** |
| *Work/School performance* | **6.01; (5.31, 6.8)** | **1.96; (1.78, 2.14)** | **2.43; (2.18, 2.7)** | **3.07; (2.71, 3.49)** | **1.24; (1.11, 1.38)** | **2.48; (2.18, 2.81)** |
| Function |  |  |  |  |  |  |
| *Antidissociation Function (H5a)* | **4.15; (3.67, 4.69)** | **2.07; (1.86, 2.29)** | **1.95; (1.75, 2.18)** | **2.01; (1.76, 2.29)** | 0.95; (0.84, 1.06) | **2.12; (1.87, 2.41)** |
| *Suicide Prevention Function (H5b)* | **4.12; (3.61, 4.71)** | **2.2; (1.98, 2.44)** | **1.95; (1.74, 2.18)** | **1.87; (1.65, 2.13)** | 0.89; (0.79, 1) | **2.11; (1.86, 2.41)** |
| Areas of injury |  |  |  |  |  |  |
| *Arms* | **3.21; (2.47, 4.16)** | **1.66; (1.39, 1.98)** | **1.79; (1.45, 2.21)** | **1.93; (1.46, 2.55)** | 1.08; (0.84, 1.38) | **1.79; (1.32, 2.42)** |
| *Chest* | **2.5; (2.16, 2.89)** | **1.57; (1.38, 1.79)** | **1.67; (1.43, 1.95)** | **1.59; (1.38, 1.83)** | 1.06; (0.91, 1.24) | **1.5; (1.3, 1.73)** |
| *Face (H6a)* | **1.27; (1.1, 1.46)** | 1.02; (0.89, 1.16) | 1.19; (1.03, 1.37) | **1.25; (1.08, 1.44)** | 1.17; (1.01, 1.35) | 1.07; (0.92, 1.25) |
| *Feet* | **2.33; (1.92, 2.82)** | **1.5; (1.24, 1.81)** | **1.51; (1.24, 1.84)** | **1.55; (1.31, 1.83)** | 1.01; (0.84, 1.21) | **1.54; (1.29, 1.84)** |
| *Hands* | **1.96; (1.72, 2.23)** | **1.25; (1.12, 1.4)** | **1.38; (1.23, 1.55)** | **1.57; (1.37, 1.79)** | 1.11; (0.98, 1.25) | **1.42; (1.24, 1.62)** |
| *Legs* | **2.01; (1.73, 2.34)** | **1.53; (1.34, 1.74)** | **1.67; (1.45, 1.92)** | **1.32; (1.11, 1.56)** | 1.09; (0.95, 1.26) | 1.2; (1.02, 1.42) |
| *Privates (H6b)* | **2.67; (2.11, 3.38)** | **1.44; (1.13, 1.85)** | **1.46; (1.1, 1.95)** | **1.85; (1.49, 2.3)** | 1.01; (0.8, 1.28) | **1.83; (1.44, 2.31)** |
| *Stomach* | **2.52; (2.21, 2.86)** | **1.56; (1.39, 1.76)** | **1.65; (1.44, 1.9)** | **1.61; (1.41, 1.84)** | 1.06; (0.93, 1.21) | **1.52; (1.32, 1.75)** |
| *Other* | **1.47; (1.25, 1.73)** | 1.13; (0.97, 1.31) | 1.18; (1.01, 1.39) | **1.3; (1.12, 1.52)** | 1.05; (0.9, 1.23) | **1.24; (1.05, 1.46)** |
| Experiences of trauma (H7) |  |  |  |  |  |  |
| *Child abuse/violence* | **1.86; (1.6, 2.17)** | **1.43; (1.25, 1.64)** | **1.36; (1.16, 1.59)** | **1.3; (1.13, 1.5)** | 0.95; (0.82, 1.09) | **1.37; (1.18, 1.6)** |
| *Death of a loved one* | **2.21; (1.88, 2.61)** | **1.34; (1.15, 1.57)** | **1.42; (1.19, 1.69)** | **1.65; (1.39, 1.95)** | 1.06; (0.9, 1.24) | **1.56; (1.32, 1.85)** |
| *Family conflict (identity/acceptance/*  *separation/divorce)* | **1.91; (1.65, 2.22)** | **1.42; (1.26, 1.61)** | **1.41; (1.21, 1.64)** | **1.34; (1.16, 1.55)** | 0.99; (0.86, 1.15) | **1.36; (1.16, 1.58)** |
| *Serious illness/injury/assault* | **3; (2.42, 3.73)** | 1.3; (1.04, 1.62) | **1.59; (1.26, 2.01)** | **2.31; (1.89, 2.83)** | 1.23; (0.99, 1.52) | **1.88; (1.54, 2.3)** |
| *Intimate partner violence* | **1.47; (1.17, 1.84)** | 1.01; (0.81, 1.27) | 1; (0.77, 1.29) | **1.45; (1.15, 1.81)** | 0.98; (0.76, 1.26) | 1.47; (1.15, 1.89) |
| *Sexual assault/violence* | **1.94; (1.67, 2.26)** | **1.31; (1.14, 1.5)** | **1.29; (1.11, 1.51)** | **1.48; (1.28, 1.72)** | 0.99; (0.85, 1.14) | **1.5; (1.28, 1.76)** |
| *Other* | 1.44; (1.07, 1.94) | **1.51; (1.18, 1.93)** | 1.05; (0.78, 1.41) | 0.95; (0.72, 1.27) | 0.7; (0.52, 0.93) | 1.37; (1, 1.88) |
| Treatment |  |  |  |  |  |  |
| *Lifetime treatment (H8a)* | **0.65; (0.57, 0.75)** | 0.97; (0.86, 1.08) | 0.86; (0.75, 0.99) | **0.68; (0.59, 0.78)** | 0.89; (0.78, 1.02) | **0.76; (0.65, 0.88)** |
| *Current treatment (H8b)* | 1.04; (0.85, 1.27) | 1.11; (0.95, 1.31) | 1.11; (0.93, 1.33) | 0.93; (0.75, 1.16) | 1; (0.81, 1.24) | 0.93; (0.75, 1.16) |
| **Other characteristics** |  |  |  |  |  |  |
| Behavior change |  |  |  |  |  |  |
| *I want to decrease my self-injury* | **0.36; (0.32, 0.41)** | **0.5; (0.45, 0.55)** | **0.79; (0.71, 0.88)** | **0.73; (0.64, 0.83)** | **1.58; (1.41, 1.77)** | **0.46; (0.41, 0.52)** |
| *I want to stop my self-injury* | **0.34; (0.31, 0.39)** | **0.48; (0.43, 0.53)** | **0.72; (0.65, 0.81)** | **0.72; (0.63, 0.82)** | **1.52; (1.35, 1.7)** | **0.48; (0.42, 0.54)** |
| *I want to stop myself-injury, but haven't been able to* | **1.29; (1.14, 1.45)** | 0.95; (0.87, 1.05) | **1.55; (1.39, 1.73)** | **1.35; (1.19, 1.53)** | **1.63; (1.45, 1.82)** | **0.83; (0.73, 0.94)** |
| Expectancies |  |  |  |  |  |  |
| *Feel good or better than I do* | **4.44; (3.92, 5.03)** | **1.96; (1.78, 2.16)** | **1.65; (1.48, 1.83)** | **2.26; (1.98, 2.58)** | **0.84; (0.75, 0.94)** | **2.7; (2.38, 3.06)** |
| *Resolve problems with others* | **4.05; (3.58, 4.59)** | **1.31; (1.2, 1.44)** | **1.84; (1.64, 2.06)** | **3.08; (2.71, 3.51)** | **1.4; (1.25, 1.57)** | **2.2; (1.95, 2.49)** |
| *Stop or relieve bad feelings or thoughts* | **2.7; (2.37, 3.08)** | **1.46; (1.33, 1.61)** | **1.5; (1.34, 1.67)** | **1.85; (1.62, 2.11)** | 1.03; (0.92, 1.15) | **1.8; (1.57, 2.07)** |

Note. Bold indicates significant at p < .01

Appendix C. Pairwise comparisons and odds ratios for hypothesized relationships controlling for age

|  | **High vs. Low** | **Moderate-SI vs. Low** | **Moderate vs. Low** | **High vs. Moderate-SI** | **Moderate vs. Moderate-SI** | **High vs. Moderate** |
| --- | --- | --- | --- | --- | --- | --- |
| **Hypothesized Relationships** |  |  |  |  |  |  |
| Duration (H1) | **2.54; (2.26, 2.86)** | **1.62; (1.46, 1.79)** | **1.67; (1.5, 1.87)** | **1.57; (1.39, 1.78)** | 1.03; (0.92, 1.17) | **1.52; (1.35, 1.72)** |
| Lifetime frequency (H2) | **4.99; (4.44, 5.61)** | **2.59; (2.35, 2.85)** | **2.45; (2.18, 2.74)** | **1.93; (1.72, 2.17)** | 0.95; (0.85, 1.06) | **2.04; (1.8, 2.31)** |
| Habituation (H3) | **9.26; (8.15, 10.53)** | **2.88; (2.6, 3.19)** | **3.11; (2.77, 3.48)** | **3.22; (2.81, 3.68)** | 1.08; (0.96, 1.21) | **2.98; (2.61, 3.41)** |
| Consequences/Life interference (H4) |  |  |  |  |  |  |
| *Enjoyable activities* | **6.95; (6.13, 7.87)** | **2.24; (2.04, 2.47)** | **2.59; (2.33, 2.88)** | **3.1; (2.74, 3.5)** | 1.15; (1.03, 1.29) | **2.68; (2.37, 3.04)** |
| *Relationships* | **3.8; (3.38, 4.27)** | **1.63; (1.48, 1.79)** | **2.08; (1.86, 2.32)** | **2.33; (2.09, 2.6)** | **1.28; (1.15, 1.42)** | **1.83; (1.62, 2.07)** |
| *Ability to care for self* | **7.46; (6.61, 8.41)** | **2.18; (1.99, 2.39)** | **2.53; (2.28, 2.81)** | **3.42; (3.02, 3.87)** | **1.16; (1.04, 1.29)** | **2.95; (2.6, 3.35)** |
| *Work/School performance* | **6.48; (5.75, 7.3)** | **2.02; (1.84, 2.21)** | **2.54; (2.29, 2.82)** | **3.21; (2.84, 3.63)** | **1.26; (1.13, 1.4)** | **2.55; (2.25, 2.89)** |
| Function |  |  |  |  |  |  |
| *Antidissociation Function (H5a)* | **4.11; (3.65, 4.62)** | **2.09; (1.88, 2.31)** | **1.97; (1.77, 2.19)** | **1.97; (1.73, 2.24)** | 0.94; (0.84, 1.06) | **2.09; (1.83, 2.37)** |
| *Suicide Prevention Function (H5b)* | **4.18; (3.68, 4.76)** | **2.24; (2.01, 2.48)** | **1.99; (1.78, 2.22)** | **1.87; (1.65, 2.12)** | 0.89; (0.79, 1) | **2.1; (1.84, 2.39)** |
| Areas of injury |  |  |  |  |  |  |
| *Arms* | **3.61; (2.81, 4.65)** | **1.79; (1.51, 2.13)** | **2.01; (1.64, 2.47)** | **2.01; (1.53, 2.65)** | 1.12; (0.88, 1.43) | **1.8; (1.33, 2.43)** |
| *Chest* | **3.34; (2.9, 3.84)** | **1.74; (1.53, 1.98)** | **1.97; (1.7, 2.29)** | **1.91; (1.67, 2.19)** | 1.13; (0.97, 1.31) | **1.69; (1.47, 1.95)** |
| *Face (H6a)* | **1.85; (1.61, 2.12)** | 1.16; (1.02, 1.32) | **1.45; (1.26, 1.66)** | **1.59; (1.38, 1.84)** | **1.25; (1.08, 1.44)** | **1.28; (1.1, 1.48)** |
| *Feet* | **3.18; (2.64, 3.83)** | **1.7; (1.41, 2.05)** | **1.82; (1.5, 2.2)** | **1.87; (1.59, 2.2)** | 1.07; (0.89, 1.29) | **1.75; (1.46, 2.09)** |
| *Hands* | **2.47; (2.18, 2.79)** | **1.36; (1.22, 1.51)** | **1.57; (1.4, 1.76)** | **1.82; (1.6, 2.07)** | 1.16; (1.03, 1.3) | **1.57; (1.38, 1.79)** |
| *Legs* | **2.36; (2.04, 2.73)** | **1.66; (1.46, 1.89)** | **1.89; (1.65, 2.18)** | **1.42; (1.2, 1.68)** | 1.14; (0.99, 1.31) | **1.24; (1.05, 1.47)** |
| *Privates (H6b)* | **3.97; (3.16, 4.99)** | **1.69; (1.32, 2.16)** | **1.86; (1.4, 2.47)** | **2.35; (1.9, 2.9)** | 1.1; (0.87, 1.39) | **2.14; (1.69, 2.71)** |
| *Stomach* | **3.22; (2.84, 3.64)** | **1.72; (1.53, 1.94)** | **1.92; (1.68, 2.2)** | **1.87; (1.64, 2.12)** | 1.12; (0.98, 1.27) | **1.67; (1.45, 1.93)** |
| *Other* | **1.79; (1.53, 2.09)** | **1.22; (1.05, 1.41)** | **1.33; (1.14, 1.56)** | **1.47; (1.26, 1.7)** | 1.1; (0.94, 1.28) | **1.34; (1.14, 1.57)** |
| Experiences of trauma (H7) |  |  |  |  |  |  |
| *Child abuse/violence* | **2.93; (2.52, 3.41)** | **1.72; (1.5, 1.98)** | **1.79; (1.52, 2.1)** | **1.7; (1.48, 1.95)** | 1.04; (0.9, 1.2) | **1.64; (1.41, 1.91)** |
| *Death of a loved one* | **3.26; (2.78, 3.83)** | **1.57; (1.35, 1.83)** | **1.8; (1.51, 2.14)** | **2.08; (1.76, 2.46)** | 1.14; (0.97, 1.35) | **1.82; (1.54, 2.14)** |
| *Family conflict (identity/acceptance/ separation/divorce)* | **2.7; (2.33, 3.12)** | **1.64; (1.45, 1.86)** | **1.75; (1.5, 2.03)** | **1.64; (1.43, 1.89)** | 1.06; (0.92, 1.23) | **1.54; (1.32, 1.8)** |
| *Serious illness/injury/assault* | **4.63; (3.75, 5.73)** | **1.52; (1.22, 1.9)** | **2.04; (1.61, 2.57)** | **3.05; (2.5, 3.72)** | **1.34; (1.07, 1.67)** | **2.28; (1.86, 2.78)** |
| *Intimate partner violence* | **2.47; (1.97, 3.1)** | 1.23; (0.98, 1.54) | 1.33; (1.03, 1.72) | **2.01; (1.6, 2.54)** | 1.09; (0.84, 1.4) | **1.85; (1.43, 2.39)** |
| *Sexual assault/violence* | **2.96; (2.56, 3.43)** | **1.56; (1.36, 1.79)** | **1.68; (1.44, 1.95)** | **1.9; (1.64, 2.19)** | 1.08; (0.93, 1.25) | **1.77; (1.51, 2.07)** |
| *Other* | **1.84; (1.38, 2.46)** | **1.66; (1.3, 2.12)** | 1.21; (0.9, 1.63) | 1.11; (0.84, 1.47) | 0.73; (0.55, 0.98) | **1.52; (1.11, 2.08)** |
| Treatment |  |  |  |  |  |  |
| *Lifetime treatment (H8a)* | 0.92; (0.8, 1.05) | 1.11; (0.99, 1.25) | 1.07; (0.92, 1.23) | **0.83; (0.72, 0.95)** | 0.96; (0.84, 1.1) | 0.86; (0.74, 1) |
| *Current treatment (H8b)* | 0.98; (0.8, 1.19) | 1.08; (0.92, 1.27) | 1.06; (0.89, 1.27) | 0.91; (0.73, 1.13) | 0.98; (0.79, 1.22) | 0.92; (0.74, 1.14) |
| **Other characteristics** |  |  |  |  |  |  |
| Behavior change |  |  |  |  |  |  |
| *I want to decrease my self-injury* | **0.39; (0.35, 0.43)** | **0.51; (0.46, 0.56)** | **0.82; (0.74, 0.91)** | **0.76; (0.68, 0.86)** | **1.61; (1.44, 1.8)** | **0.47; (0.42, 0.54)** |
| *I want to stop my self-injury* | **0.37; (0.33, 0.42)** | **0.49; (0.44, 0.54)** | **0.75; (0.67, 0.84)** | **0.76; (0.67, 0.86)** | **1.54; (1.37, 1.73)** | **0.49; (0.44, 0.56)** |
| *I want to stop myself-injury, but haven't been able to* | **1.39; (1.24, 1.57)** | 0.98; (0.89, 1.08) | **1.62; (1.45, 1.81)** | **1.42; (1.26, 1.6)** | **1.65; (1.48, 1.85)** | 0.86; (0.76, 0.97) |
| Expectancies |  |  |  |  |  |  |
| *Feel good or better than I do* | **4.53; (4.01, 5.11)** | **1.99; (1.81, 2.19)** | **1.68; (1.52, 1.87)** | **2.27; (2, 2.59)** | **0.84; (0.75, 0.94)** | **2.69; (2.38, 3.05)** |
| *Resolve problems with others* | **4.02; (3.57, 4.53)** | **1.31; (1.2, 1.44)** | **1.84; (1.65, 2.05)** | **3.06; (2.7, 3.47)** | **1.4; (1.25, 1.57)** | **2.18; (1.93, 2.46)** |
| *Stop or relieve bad feelings or thoughts* | **2.66; (2.34, 3.02)** | **1.46; (1.33, 1.61)** | **1.5; (1.35, 1.67)** | **1.82; (1.6, 2.08)** | 1.03; (0.92, 1.15) | **1.77; (1.55, 2.04)** |

Note. Bold indicates significant at p < .01
